# Evaluation of fatty liver fibrosis in rabbits using real-time shear wave elastography

**DOI:** 10.3892/etm.2014.1749

**Published:** 2014-05-29

**Authors:** YONGPING LU, JIA WEI, YUEYUE TANG, YUAN YUAN, YANLING HUANG, YONG ZHANG, YUNYAN LI

**Affiliations:** 1 Department of Ultrasound, The Fourth Affiliated Hospital of Kunming Medical University, The Second People’s Hospital of Yunnan Province, Kunming, Yunnan 650021, P.R. China; 2 The Liver Disease Center, The Fourth Affiliated Hospital of Kunming Medical University, The Second People’s Hospital of Yunnan Province, Kunming, Yunnan 650021, P.R. China

**Keywords:** real-time shear wave elastography, rabbit, fatty liver, elasticity

## Abstract

The aim of the present study was to detect the elastic modulus (stiffness) of the livers of rabbits with non-alcoholic and alcoholic fatty liver disease using real-time shear wave elastography (SWE), and to investigate the fibrosis development process in the formation of fatty liver. The stiffness of the fatty livers in rabbit models prepared via feeding with alcohol or a high-fat diet were measured using a real-time SWE ultrasound system and a 4–15-MHz linear array probe, and the liver stiffness was compared with the pathological staging of the disease. The stiffness of the liver was positively correlated with the degree of pathological change in fatty liver disease (P<0.01). The stiffness of the liver in the alcoholic fatty liver group was higher compared with that in the non-alcoholic fatty liver and control groups, and the stiffness in the non-alcoholic fatty liver group was higher than that in the control group (P<0.01). Real-time SWE objectively identified the trend in the changing stiffness of the liver and noninvasively detected the development of fibrosis in the progression of non-alcoholic and alcoholic fatty liver disease.

## Introduction

As the lifestyle of the population worldwide changes, fatty liver disease is gradually increasing in incidence (1) and has become the second most severe type of liver disease after viral hepatitis (2). Although fatty liver disease is considered to be a benign disease, without treatment it gradually develops into inflammatory cell infiltration and necrosis. This results in liver fibrosis and cirrhosis and may lead to a malignant disease, such as hepatocellular carcinoma (3). Histopathological examination via a liver puncture is considered to be the gold standard for evaluation of the risk and the severity of fatty liver disease (4–7). However, as it is an invasive examination, liver puncturing is not readily accepted by patients; therefore, an appropriate imaging examination is the preferred method for evaluating the severity of fatty liver disease. Traditional ultrasound exhibits a strong subjectivity in the quantitative diagnosis of fatty liver disease, whereas computed tomography, magnetic resonance imaging and other imaging techniques pose numerous limitations in the evaluation of fatty liver disease. Thus, studies to identify a noninvasive technology for the diagnosis of fatty liver disease are required (3,8).

The ultrasound elastography method objectively demonstrates elasticity information of tissue and reflects the stiffness of the measured tissue using grayscale or color images (9,10). The real-time shear wave elastography (SWE) method that was developed on the basis of this, rapidly, noninvasively, objectively and quantitatively detects the degree of fibrosis of liver diseases, including fatty liver disease, and has attracted increasing attention (10).

The present study investigated a rabbit model using the real-time SWE technique and investigated the fibrosis development process associated with the progression of non-alcoholic and alcoholic fatty liver disease to provide objective indices for clinical intervention and to facilitate the evaluation of curative effects.

## Materials and methods

### Animal grouping

Thirty male Japanese white rabbits (age, 10–26 days; body weight, 0.7–1.52 kg) were randomly divided into three groups of 10 rabbits per group. One group was a rabbit model of simple fatty liver disease obtained via high-fat diet feeding (formula; 2% cholesterol, 10% butter, 5% white sugar, 8% egg yolk powder and 75% conventional, basal feed) and water intake was *ad libitum*. This process was conducted by the Laboratory Animal Research Center of Kunming Medical University (Kunming, China). The second group was a rabbit model of alcoholic fatty liver disease obtained via conventional feeding (basal feed), a 10 ml infusion of Chinese spirits [Chinese spirits (Beijing Red Star CO., Ltd., Beijing, China)] twice a day and water intake *ad libitum*. The feeding method was as follows: 10 ml spirit was injected into the pharynx oralis of the rabbit with the mouth forced shut for 2 min until the rabbit swallowed. Care was taken to ensure that there was no leakage of the spirit from the mouth. The alcoholic beverage comprised drinking water and alcohol at a concentration of 15% ethanol, which was provided by the Animal Laboratory of Kunming Medical University (Kunming, China). The third group served as a normal control group with conventional feeding (basal feed). After feeding for 12 weeks, the rabbits were fasted for 12 h, weighed and sacrificed following performance of ultrasonography and elastography. The animals were humanely sacrificed by air embolism method. After obtaining the weight of the liver, it was pathologically observed. The present study was conducted in strict accordance with the recommendations set out in the Guide for the Care and Use of Laboratory Animals of the National Institutes of Health (eighth edition, 2011). The animal use protocol was reviewed and approved by the Institutional Animal Care and Use Committee of the Fourth Affiliated Hospital of Kunming Medical University (Kunming, China).

### Ultrasonography

Real-time SWE studies were performed using the Aixplorer^®^ ultrasound system (SuperSonic Imagine S.A., Aix-en-Provence, France) with a linear array probe (L15-4) and a frequency of 4–15 MHz.

The rabbits were fixed in the supine position without anesthesia. The skin was prepared from the fourth right rib to the abdomen so as to fully expose the area to be examined. The abdomen and liver were initially subjected to comprehensive and systematic ultrasonography, including identification of the size, echo, capsule and ascites of the liver. The mode was switched to SWE, the probe was gently moved without exerting any pressure and once the image was stable, the frame was determined. The elasticity in the selected region of interest was measured using the method provided by the ultrasonic apparatus (vessel structures were avoided during sampling). For sampling, two segments of the hepatic right lobe in the longitudinal section of the first hepatic portal (including the junction of the portal vein, proper hepatic artery and the common bile duct) and one segment of the left lobe in the xiphoideusal horizontal section were selected; three samples were taken from each segment. When the deviation of the values determined for the three samples was <10%, the sampling was considered to be successful. Mean values were calculated for each segment, and the mean value of the means of all the segments was calculated to obtain the required data. Data determined for each group were the maximum elastic modulus (Max), the mean elastic modulus (Mean), the minimum elastic modulus (Min) and the speed dispersion (Sd). The data were measured automatically using an Aixplorer ultrasound system, and the images and data were recorded and saved on a computer.

Max reflected tissue structures with the highest stiffness within a sampling area, including diseased tissue and certain structures in the liver, such as ligaments, whereas Min reflected tissue structures with the lowest stiffness in a sampling area, including the lumens of the intrahepatic blood vessels and the bile duct. Mean was the mean elastic modulus of the entire area and the standard deviation reflected the uniformity of the tissue stiffness in the sampling area, and the successful sampling rate.

### Liver sample collection and gross observation

On completion of the ultrasonography, the rabbits were sacrificed for removal of the liver, which was weighed and observed for size, shape, color, surface smoothness, interlobar fissure width, edge sharpness and composition. The liver tissue samples were immobilized with 10% formalin, followed by routine dehydration, vitrification, waxing, embedding, slicing (thickness, 4–6 μm), hematoxylin and eosin (H&E) staining, and picrosirius red staining (Shanghai Jianglai Biotechnology Co., Ltd., Shanghai, China).

The degree of pathological change of fatty liver disease in the rabbits was observed under a microscope (Nikon Eclipse E200, Tokyo, Japan) and divided into five degree classes according to the area of steatosis that was observed in the liver cell specimen under the light microscope (11,12). Normal liver F0, steatosis area <10%; slight steatosis F1, steatosis area 10–33%; mild fatty liver F2, steatosis area 33–50%; moderate fatty liver F3, steatosis area 50–66%; and severe fatty liver F4, >66% steatosis of liver cells.

### Picrosirius red and silver staining

A paraffin section was successively dewaxed using xylene, and washed and stained in 0.5% picrosirius red for 5–30 min, washed with distilled water three times, differentiated with absolute ethanol and dehydrated. Observation under a light microscope demonstrated that the collagen fibers were red, the nucleus was green and the other components were yellow.

The sections were dewaxed and then oxidized with 0.25% potassium permanganate solution. After washing with water, the sections were bleached using 2% oxalic acid solution and washed with water again. The sections were then mordanted with a 2% aqueous solution of ammonium sulfate and washed with water, prior to staining with Gomori silver ammonia solution, washing with water, deoxidizing with 4% neutral formalin solution and washing with water. After toning with 0.2% gold chloride and washing with water, the sections were fixed with 5% sodium thiosulfate, conventionally dehydrated until transparent, and sealed and cemented with neutral vegetable glue.

### Staging

The staging was determined according to the formation of fibrous tissue (13) as follows: Stage (S) 0, normal liver without fibrosis; S1, fibrosis formed in a small region, predominantly including the fibers in and around the portal area; S2, a plurality of fibrous septa, with the lobular structure being roughly retained; S3, a large quantity of fibrous septa, causing the lobular structure to be disordered but without cirrhosis; S4, early cirrhosis.

### Statistical analysis

Student’s t-test of the various indices of the liver in the three groups was performed using SPSS statistical software, version 13 (SPSS Inc., Chicago, IL, USA) with all the data displayed as means ± standard deviation. A correlation analysis between the Mean and the area of pathological steatosis was performed. P<0.05 was considered to indicate a statistically significant result.

## Results

### General data

In the alcohol feeding group, three rabbits died at 10 weeks old. The anatomical results showed apparent ascites due to cirrhosis and serious liver damage, and the deaths were ascribed to liver damage and malnutrition. In the normal group, one rabbit died at nine weeks due to diarrhea, rather than loss of hepatic function; liver samples were collected from the rabbit and the results showed normal liver. By contrast, the rabbits in the high-fat diet feeding group all survived. Ten rabbits with a non-alcoholic fatty liver and seven rabbits with an alcoholic fatty liver were successfully established as models 12 weeks after ultrasonography.

### Ultrasonography of the liver in the three groups

The livers of the nine normal rabbits exhibited an echo marginally lower than a moderate level. The internal echo was identified to be uniform, the light spots were moderately intense, the vessel structure was clear and the echo of the vessel wall was stronger than that of the liver parenchyma. Furthermore, the capsule was regular and smooth without exhibiting ascites.

The 10 rabbit models with simple fatty liver exhibited an enlarged liver with an increased echo, a significant attenuation in the frontal region, partial attenuation in the posterior region and fine light spots. The vessel structure was less clear than that of the normal group. The echo of the vessel wall was enhanced, although not as markedly as that of the liver parenchyma, and the capsule was predominantly smooth. One rabbit presented with ascites, which were indicated by a dark area of 1–3 cm diameter in the space between the liver and the kidney.

**Figure 1:**
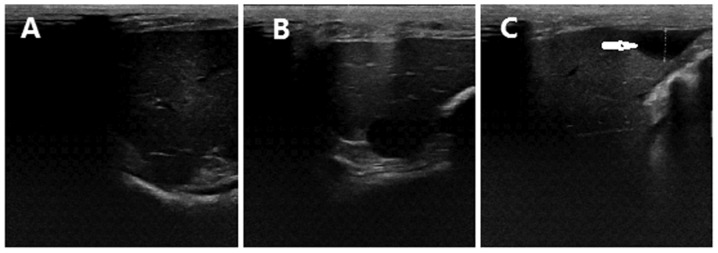
Two-dimensional images of rabbit livers. (A) Normal rabbit liver. (B) Simple fatty liver. (C) Alcoholic fatty liver (the scale bar indicates ascites).

The seven rabbit models with alcoholic fatty liver exhibited narrowing of the liver. The ultrasonography results were comparable to those of the fatty liver group, however, their degree of variation was more apparent. Two rabbits demonstrated a less regular liver capsule and exhibited ascites to different degrees and the ultrasonography demonstrated cirrhosis (Fig. 1 and Table I).

### Elastic modulus of the three groups and a comparison of the related data

**Figure 2:**
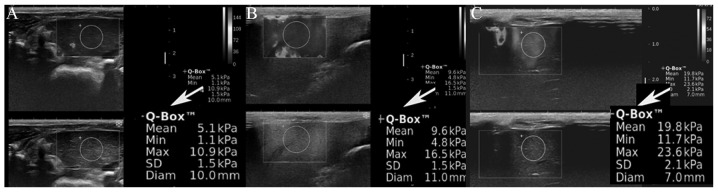
Elastography of the rabbit liver. Mean elastic modulus of: (A) the liver in a normal rabbit, 5.1 kPa; (B) a non-alcoholic fatty liver, 9.6 kPa; (C) an alcoholic fatty liver, 19.8 kPa.

The age of the rabbits in the three groups was not significantly different (P>0.05). Liver elasticity was measured and demonstrated that the sampling volume of the region of interest in the liver of the three groups of rabbits was not significantly different (P>0.05). The Mean, Max and Min reflected the liver stiffness and showed the following: The values for the alcoholic fatty liver group were higher than those in the simple fatty liver group and the normal group, and the values in the simple fatty liver group were higher than those in the normal group (P<0.05; Fig. 2 and Table I).

### Observation of the gross liver samples of the rabbits in the three groups

**Figure 3:**
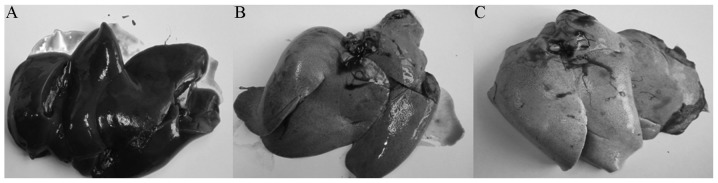
Gross samples of rabbit livers. (A) Normal liver. (B) Non-alcoholic fatty liver. (C) Alcoholic fatty liver.

After the rabbits were sacrificed, the gross liver samples of the rabbits were observed. The color of the livers in the nine normal rabbits was dark red and the left lobe was significantly larger than the right lobe. Compared with the normal group, the fatty liver groups exhibited stiffer livers, with a moderately hard, elastic texture. The liver had good elasticity and the interlobar fissure was clear with a sharp edge and smooth section. The livers of the 10 rabbits with non-alcoholic fatty liver disease were identified to be uniformly enlarged with full hepatic lobes. The right lobe was enlarged and the ratio of left lobe size to right lobe size was reduced; furthermore, the interlobar fissure became smaller and the liver edge became blunt. The livers were a gray-red color and were stiffer than those of the normal rabbits with a moderately stiff and elastic texture. Of the seven rabbits, five exhibited enlarged livers, with a morphological structure that was comparable to that observed in the simple fatty liver group. Two of the livers were significantly reduced in volume, with an uneven surface and exhibited an indistinct or concealed interlobar fissure. The size of the left lobe was identified to be equal to that of the right one. Compared with the other groups, the alcoholic fatty liver group displayed noticeably stiffened livers that were hard in texture. The livers were gray-white in color with a granular section and when the liver was being sectioned, it was stiff and sounded as though fibrous tissue was being cut (Fig. 3).

### Observation of the livers from the three groups of rabbits using a microscope

**Figure 4:**
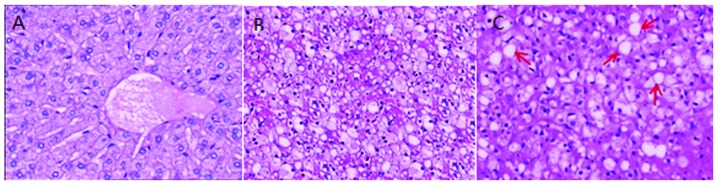
Hematoxylin and eosin staining of the rabbit liver observed under a microscope (magnification, ×200). (A) Liver from a normal rabbit; liver cell cords arranged in order with occasional lipid droplets in the liver cells. (B) Non-alcoholic fatty liver; a large number of large-droplet liver cells were apparent, which accounted for ~50% of the total area. (C) Alcoholic fatty liver; large-droplet liver cells accounted for ~80% of the total area, the foam-like cells are indicated by red arrows.

The lobules of the liver that were obtained from the rabbits in the normal group displayed a complete structure and the hepatic cords were normal. Lipid droplets were occasionally observed in the liver cells and 5.3–8.9% of the area of the liver displayed steatosis. Lipid droplets of a uniform size (diameter, 3–5 μm) were scattered in the liver cells of the rabbits with simple fatty liver. These lipid droplets may have fused and undergone nuclear deviation or centered around the nucleus, and the area of steatosis was identified to constitute 25–55% of the liver. The livers of the rabbits in the alcoholic fatty liver group were suffused with lipid droplets in a panlobular manner. The liver cells were swollen, the sinus space was narrow, and the nuclei and organelles moved to the borders of cells due to the extrusion of the lipid droplets; in addition, foam-like cells were observed. The liver cells were 4–5 times larger than the normal liver cells, the intracellular structure was not able to be identified and the area of steatosis accounted for 60–80% of the total area of the liver (Fig. 4).

**Figure 5:**
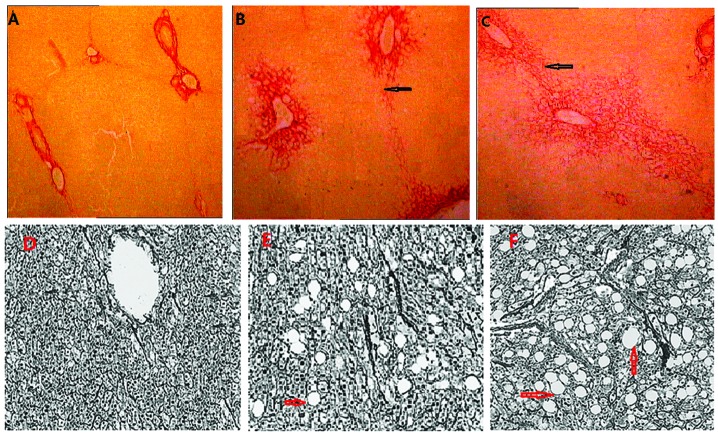
Picrosirius red (A–C) and silver (D–F) staining of the rabbit liver (magnification, ×200). Microscope images: (A) Normal rabbit liver, a small number of fibrous tissues were observed in the portal area. (B) Non-alcoholic fatty liver, fibrosis occurred around the portal area with fibrous septa forming in the lobule. (C) Alcoholic fatty liver, a large number of fibrous septa formed, which damaged the hepatic lobule. The red structures indicated by the arrows are fibrous tissues. (D) Normal rabbit liver, a small number of fibrous tissues were observed. (E) Non-alcoholic fatty liver, increased fibrosis occurred. (F) Alcoholic fatty liver, a large number of fibrous tissues were observed.

Picrosirius red staining indicated the following: There was no evident fibrosis in the livers of the rabbits in the normal group (all nine rabbits were S0); the non-alcoholic fatty liver group predominantly showed inflammation and fibrosis formation in the portal area, including three rabbits at S1, six rabbits at S2 and one rabbit at S3; the alcoholic fatty liver group predominantly exhibited fibrous septa with lobular structure disorders, including one rabbit at S1, two rabbits at S2, two rabbits at S3 and two rabbits at S4 (the cirrhosis stage; Fig. 5 and Table II).

**Figure 6:**
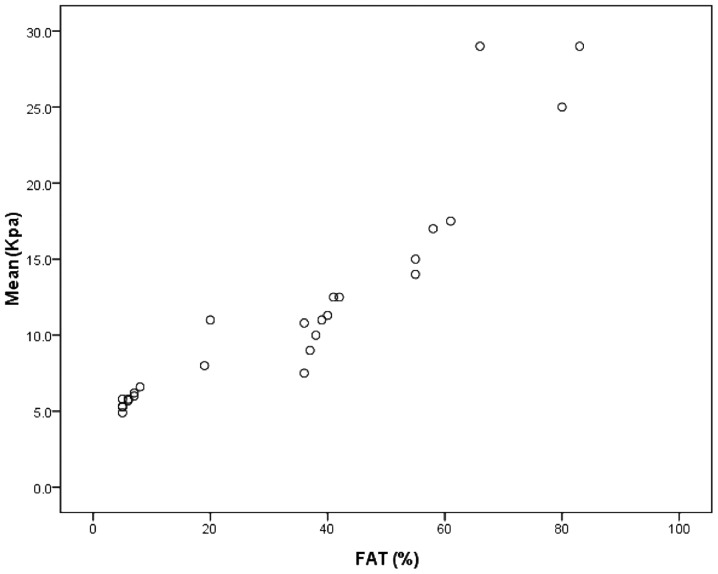
Correlation analysis indicated that the mean elasticity of the rabbit liver was positively correlated with the steatosis area (r=0.92, P<0.01).

**Figure 7:**
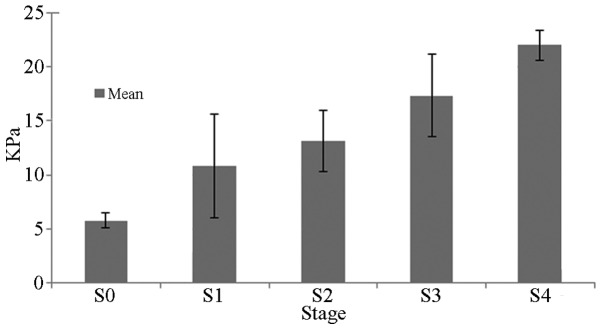
Correlation between the mean of the mean elastic modulus values and the stage. Significant differences were observed among the different groups (P<0.05). Stage S0, normal liver essentially without fibrosis; S1, fibrosis formed in a small region, predominantly including fibers in and around the portal area; S2, a plurality of fibrous septa, where the lobular structure is roughly retained; S3, a large quantity of fibrous septa with a disordered lobular structure but without cirrhosis; S4, early cirrhosis.

The correlation analysis indicated that the Mean of the liver in the 26 rabbits (all three groups) was positively correlated with the pathological steatosis area (r=0.92, P<0.01), as was the Max (r=0.67, P<0.05). Furthermore, Mean and Max were identified to be positively correlated with the steatosis area between S1 and S3 (P<0.05; Table III and Fig. 6). The analysis of variance results of the 26 rabbits at the different fibrosis stages (S0–4) indicated statistically significant differences among the Mean values, and also among the Max values, for the different stages. The Mean values were as follows: S0, 5.78±0.66 kPa; S1, 10.83±4.81 kPa; S2, 13.15±2.82 kPa; S3, 17.33±3.79 kPa; and S4, 22.0±1.41 kPa (Table III and Fig. 7). The results indicated that a higher stage implied a greater elastic modulus (stiffness).

## Discussion

The application of ultrasound elastography in the screening of patients with fatty liver disease is able to identify at an early stage whether the liver is exhibiting fibrosis, which facilitates the monitoring of fatty liver disease and aids clarification of the pathological type.

Ultrasound elastography is a technique that reflects the tissue hardness via digital imaging (14–16) and SWE, which is derived from this technique, directly measures the Young’s modulus (elastic modulus) of tissues (8,17).

The ultrasonic diagnostic apparatus widely used for instantaneous clinical elastography in the early stages of disease includes the French FibroScan^®^, which has been used for studies of viral hepatitis (B and C) as well as for the quantitative diagnosis of liver fibrosis. However, it is a one-dimensional instantaneous elastography system and adipose tissue markedly attenuates the low frequency shear and ultrasonic waves; therefore, the application of this apparatus is limited during observation of fatty liver disease (18).

The use of Aixplorer (10,19,20) real-time SWE achieves two-dimensional color imaging with a maximum imaging depth of 14 cm, overcomes the attenuation of the adipose tissue on sound velocity and is, therefore, used to observe fatty liver stiffness. A certain study has demonstrated that liver stiffness is well correlated with the stage of liver fibrosis (21).

Thus, the present study simulated the current poor diet of humans and established an animal model of fatty liver disease by administering a high-fat diet and alcoholic drinks, respectively and studied the models using conventional ultrasonography and real-time SWE using Aixplorer ultrasound system.

The present study indicated, via continuous ultrasonic observation, that all the rabbits that were fed a high-fat diet exhibited fatty liver disease 12 weeks later, confirming that the model was successfully established. Furthermore, the present study investigated the disease features and pathological quantitative diagnosis indices of fatty liver disease. Generally, fatty liver disease develops from a simple fatty liver to steatohepatitis and eventually to fatty cirrhosis (3,20). However, there are exceptions, for example, alcoholic fatty liver may not exhibit clear steatohepatitis, but directly evolve to cirrhosis (3). The animal models of fatty liver disease (non-alcoholic and alcoholic) that were established in the present study via different feeding methods also followed the abovementioned processes. However, as rabbits are grazing animals and have a poor lipid metabolic capability, a high-fat diet may have resulted in a rapid progression of fatty liver disease and severe injury. They rapidly entered the stages of steatohepatitis, fibrosis and cirrhosis and similarly developed inflammatory cell infiltration and fibrogenesis.

Ultrasonography demonstrated that the majority of the rabbits in the high-fat diet group developed moderate fatty liver disease and seldom developed cirrhosis. By contrast, the rabbits in the alcoholic diet group exhibited noticeable liver lesions; the majority developed severe fatty liver disease and the number of cases of cirrhosis increased. These results were further evidenced via a pathological examination; compared with the high-fat diet group, the alcoholic diet group indicated greater areas of hepatic steatosis (25–55% versus 60–80%; P<0.05) and more severe fibroplasia. Furthermore, the three groups of rabbits were of similar ages, however, their body weights were markedly different (high fat>normal>alcohol group), which indicated that the growth of the rabbits in the alcohol group was restricted and significantly associated with the liver injury.

In addition, the present study investigated the Mean and Max of the livers of the rabbits in the normal, high-fat and alcohol feeding group using real-time SWE. The results indicated that the liver stiffness in the alcoholic fatty liver group was significantly higher than that observed in the simple fatty and normal groups, and the liver stiffness in the simple fatty liver group was higher than that observed in the normal group; this coincided with the trend of the pathological changes.

All of the rabbit models underwent ultrasonography and pathological comparison, in addition to H&E and picrosirius red staining, respectively. Following H&E staining, the lipid droplets that were observed in the liver cells were examined under an optical microscope. The fatty liver was categorized into stages F0–4 according to the proportion of liver cells that exhibited steatosis in the liver tissue sections.

Sirius red is a long, unfolded molecule that undergoes an extremely stable adsorption reaction with collagen molecules, does not readily fade following staining and has strong specificity. Therefore, it is currently the optimal staining method for collagen fibers, enabling clear observation of the fibrous tissue in the liver for qualitative or quantitative diagnosis of liver fibrosis. Liver fibrosis is divided into stages S1–4 according to the deposition site and scope of the collagen fibers, the damage to the liver structure and its influence on the hepatic microcirculation (8).

Although the present study indicated that the hepatic steatosis area and the fibrosis grade positively correlated with the elastic modulus of the liver, the authors of the present study hypothesize that the latter was the key influential factor of liver stiffness, which was consistent with previous studies (18,22). The development of fatty liver disease proceeds from a simple fatty liver to steatohepatitis, fibrosis and liver cirrhosis (22). Accordingly, the liver stiffness demonstrates different variation trends at each individual stage; at the simple fatty liver stage, the liver is comparatively soft due to the lipoid degeneration of regions within the cells. At the steatohepatitis stage, the liver hardness value is normal or marginally increased as a result of cell lipoid degeneration and inflammation. At the fibrosis and liver cirrhosis stages, liver stiffness increases due to fibroplasia. In the present study, the fatty liver models were obtained through adverse factor intervention and when compared with naturally developing fatty liver disease, these models showed rapid progression. As a result of this, the majority of the models had entered the fibrosis or cirrhosis stage when the pathological examination was performed and the simple fatty liver, and occasionally the steatohepatitis stage, was not observed. Therefore, the models used in the present study failed to represent all the stages in the progression and the complex variations of human fatty liver disease. Hepatocellular fatty degeneration closely correlates with the development and severity of fatty liver disease. It becomes increasingly apparent at the advanced stages (fibrosis and cirrhosis), which indicates serious fatty liver pathological changes, a progressively noticeable fibrosis trend and a higher liver stiffness value. In addition, the present study showed that the hepatic steatosis area and liver stiffness were associated with the severity of fibrosis, identifying a positive correlation between them (r=0.92, P<0.01).

The present study had certain limitations. The assessment of the liver elasticity and pathology of fatty liver disease in the rabbits was conducted following formation of the fatty livers. Therefore, the present study lacks data regarding the formation and development of fatty liver disease. In addition, the present study simulated liver changes that were observed in rabbits resulting from bad dietary habits. As rabbits are herbivorous animals, they exhibit a different capability to humans regarding high lipid and alcohol metabolism. Therefore, the established fatty liver models in the present study only generally, rather than completely, reflect the trend of the development of fatty liver disease in humans. Thus, studies on human-related diseases are required.

In conclusion, real-time SWE exhibits satisfactory repeatability and stability in quantitatively determining liver elasticity. Furthermore, the technology is noninvasive. These advantages indicate that real-time SWE is a method worthy of wider use in clinical practice.

## Figures and Tables

**Table I:** Comparison of the measured elasticity of the rabbit livers in the three groups.

Liver type	Elastic modulus (kPa)					Diameter (mm)

	Rabbits (n)	Age (days)	Max	Min	Mean	
Normal	9	96.1±4.4	16.21±4.79	1.97±1.37	5.78±0.66	9.48±0.96
Simple fatty	10	97.2±4.9	25.73±5.21^a^	3.81±1.49^a^	10.60±2.06^a^	9.24±1.24
Alcoholic fatty	7	95.1±4.5	41.11±4.67^a^,^b^	8.42±1.76^a^,^b^	19.43±2.13^a^,^b^	9.90±1.37

a P<0.05 compared with the normal liver group;

b P<0.05 compared with the simple fatty liver group.

The various indices of the rabbits in the three groups were analyzed using Student’s t-test. Sd, speed dispersion.

**Table II:** Comparison of the pathological observations of the rabbit livers in the three groups.

Liver type	Rabbits (n)	BW (kg)	LW (kg)	Steatosis area (%)	S1 (n)	S2 (n)	S3 (n)	S4 (n)
Normal	9	2.8±0.54	0.123±0.021	6.5±2.5	0	0	0	0
Simple fatty	10	3.2±0.48^a^	0.197±0.027^a^	40.2±8.3^a^	3	6	1	0
Alcoholic fatty	7	2.5±0.67^a^,^b^	0.108±0.029^b^	75.0±10.6^a^,^b^	1	2	2	2

a P<0.05 compared with the normal liver;

b P<0.05 compared with the simple fatty liver.

BW, body weight; LW, liver weight; Stage S1, fibrosis formed in a small region, predominantly including fibers in and around the portal area; S2, a plurality of fibrous septa, where the lobular structure is roughly retained; S3, a large quantity of fibrous septa with a disordered lobular structure but without cirrhosis; S4, early cirrhosis.

**Table III:** Association between the elastic modulus and steatosis area of the rabbit liver according to fibrosis stage.

Fibrosis stage	Rabbits (n)	Steatosis area (%)	Elastic modulus (kPa)		

			Mean	Max	Min
0	9	6.5±2.5	5.78±0.66 (r=0.34, P>0.05)	6.21±4.79 (r=0.37, P>0.05)	1.97±1.37 (r=0.167, P>0.05)
1	4	32.3±7.4	10.83±4.81 (r=0.69, P<0.05)	15.62±4.31 (r=0.65, P<0.05)	3.81±1.49 (r=0.27, P>0.05)
2	8	45.8±8.1	13.15±2.82 (r=0.78, P<0.05)	25.14±5.28 (r=0.71, P<0.05)	4.42±1.76 (r=0.35, P>0.05)
3	3	64.5±9.8	17.33±3.79 (r=0.75, P<0.05)	35.23±4.99 (r=0.66, P<0.05)	5.97±1.37 (r=0.25, P>0.05)
4	2	80±8.9	22.0±1.41 (r=0.41, P>0.05)	43.73±6.56 (r=0.39, P>0.05)	4.81±1.49 (r=0.16, P>0.05)
Total	26	40.9±10.3	11.40±5.65a (r=0.92, P<0.01)	24.36±5.34a (r=0.67, P<0.05)	4.53±1.65 (r=0.16, P>0.05)

a P<0.05 compared with stages 0, 1, 2 and 3.

Analysis of variance indicated significant differences among the fibrosis stage groups; r, the correlation coefficient of elasticity and steatosis area; Stage S0, normal liver essentially without fibrosis; S1, fibrosis formed in a small region, predominantly including fibers in and around the portal area; S2, a plurality of fibrous septa, where the lobular structure is roughly retained; S3, a large quantity of fibrous septa with a disordered lobular structure but without cirrhosis; S4, early cirrhosis.
